# In Vivo Evaluation of *Cannabis sativa* Full Extract on Zebrafish Larvae Development, Locomotion Behavior and Gene Expression

**DOI:** 10.3390/ph14121224

**Published:** 2021-11-25

**Authors:** Rosario Licitra, Marco Martinelli, Luigi Petrocchi Jasinski, Maria Marchese, Claudia Kiferle, Baldassare Fronte

**Affiliations:** 1 Molecular Medicine and Neurobiology—ZebraLab, IRCCS Fondazione Stella Maris, 56128 Pisa, Italy; rosario.licitra@fsm.unipi.it; 2 PlantLab, Institute of Life Sciences, Scuola Superiore Sant’Anna, 56124 Pisa, Italy; marco.martinelli@santannapisa.it (M.M.); claudia.kiferle@santannapisa.it (C.K.); 3 Department of Veterinary Science, University of Pisa, 56124 Pisa, Italy; luigipetrocchijasins@libero.it

**Keywords:** cannabinoids, cannabis, *Cnr1*, *Cnr2*, drugs, gene expression, locomotion, locomotor performances, nutrition, zebrafish

## Abstract

Historically, humans have been using *Cannabis sativa* for both recreational and medical purposes. Nowadays, cannabis-based products have gained scientific interest due to their beneficial effects on several syndromes and illnesses. The biological activity of cannabinoids is essentially due to the interaction with the endocannabinoid system, and zebrafish (*Danio rerio*) is a very well-known and powerful in vivo model for studying such specific interactions. The aim of the study was to investigate the effects of different doses of a *Cannabis sativa* whole extract [dissolved in dimethyl sulfoxide (DMSO)] on zebrafish eggs’ hatchability, embryo post-hatching survival, larvae locomotion behavior and mRNA gene expression. The results showed the absence of toxicity, and no significant differences were observed between treatments for both embryo hatching and survival rate. In addition, larvae exposed to the cannabis extract at the highest dose [containing 1.73 nM and 22.3 nM of ∆^9^-tetrahydrocannabinol (THC) and cannabidiol (CBD), respectively] showed an increased locomotion compared to the control and DMSO treated groups. Moreover, qRT-PCR analysis showed that the highest dosage of cannabis induced an over-expression of *cnr1* and *cnr2* cannabinoid receptors. In conclusion, the exposition of zebrafish larvae to the whole extract of *Cannabis sativa* showed no negative effects on embryo development and survival and enhanced the larvae’s locomotor performances. These findings may open up possible *Cannabis sativa* applications in human pharmacology as well as in other animal sectors.

## 1. Introduction

For thousands of years, humans have been using *Cannabis sativa* as a recreational and medicinal drug. To date, about 500 natural active compounds have been identified in this powerful plant [1]. These components are mainly represented by phytocannabinoids, terpenes, terpenoids and flavonoids, as well as fatty acids, proteins, carbohydrates, chlorophylls, vitamins and minerals, among others [2]. The two main phytocannabinoids found in cannabis are ∆^9^-tetrahydrocannabinol (THC), considered a psychoactive component, and cannabidiol (CBD), which lacks psychotropic activity [3]. Precursors of these two compounds are tetrahydrocannabinolic acid (THCA) and cannabidiolic acid (CBDA), respectively [4]. THCA and CBDA can be non-enzymatically converted to THC and CBD within the plant, even if the decarboxylated forms are mainly generated by heating the harvested material [5].

Noticeably, the quantitative and qualitative components of the cannabis flowers are highly variable, since their composition, concentration and yield are greatly affected by plant genotype and growing conditions [6]. Cannabis flowers have been consumed for recreational reasons but also to relieve pain conditions, to stimulate the appetite and to fight anxiety and insomnia [7,8]. A recent survey on cannabis consumers in the United States showed that cannabis use was higher in unmarried males of 18–25 years old [9].

Nowadays, several products and cannabis extracts have been developed and studied due to their beneficial effects on animal and human behavior as well as for their therapeutic properties on several syndromes and illnesses. For instance, cannabis have been used to treat Dravet syndrome [10], multiple sclerosis [11], epilepsy [12], anxiety [13], schizophrenia [14], chronic pain [15], glaucoma [16], musculoskeletal problems such as fibromyalgia [17] and also some types of cancer [18]. The use of cannabis for medicinal purposes is rapidly spreading as a consequence of the increasingly legalization of the plant [19].

In veterinary medicine, many clinicians successfully adopted cannabis derivatives for pain control, idiopathic epilepsy refractory to conventional treatments, allergic skin diseases and mood disorders. Moreover, the use of cannabis derivatives seems not associated with the occurrence of side effects in animals, even when supplied for long periods [2]. Other cannabis applications on farm animals have been taken under consideration to cope with stressful rearing conditions (e.g., high stocking density) and practices (e.g., transportation) [20,21,22]. Unfortunately, cannabis consumption has been associated with different detrimental effects on nervous, cardiovascular, and respiratory systems [23]. Indeed, cannabinoids may have serious and undesirable effects such as dependency, psychotic illness, and cognitive impairment, including deleterious effects on memory [24].

From a biochemical point of view, cannabinoids are highly lipophilic substances and may actually remain associated with the cell membranes for long time after exposure, exerting their effects even after the direct exposure has ended [25].

The biological activities of phytocannabinoids are essentially linked to their ability to interact with the endocannabinoid system (ECS), represented by the ensemble of cannabinoid receptors, endocannabinoids (compounds produced by the body that bind to the aforementioned receptors), enzymes responsible for their metabolism and genes coding for these proteins [26]. *In vitro* studies have already demonstrated that cannabinoids play an important role on locomotor activity. In fact, using a specific antagonist (AM-251) for blocking the cannabinoid receptors 1 (CB1) in the isolated spinal cord of the lamprey (*Lampetra fluviatilis*), the baseline frequency of the locomotor rhythm was reduced. On the contrary, the activation of CB1 by endocannabinoids seems to increase the excitability in the spinal circuitry, accelerating the locomotor behavior, suggesting that endocannabinoids contribute to the expression of the motor pattern [27].

To investigate the effects of cannabinoids’ administration for preclinical drug screening, in vivo studies using effective animal models are mandatory. Phylogenetic analysis has already proved that the ECS is highly conserved between vertebrates [28], and it has been demonstrated that ECS plays a crucial role in embryogenesis and in central nervous system (CNS) development [29]. In particular, the ECS is involved in axonal growth and neural cell development [30]. In vertebrates, cannabinoids exert their neuromodulator effects through interactions with another receptor: the cannabinoid receptors 2 (CB2) [31]. The CB1, encoded by the *cnr1* gene, is expressed primarily in the CNS, and it is considered one of the most abundant G-protein-coupled receptors in the brain [30]. In humans, the high levels of CB1 in basal ganglia, cerebral cortex and cerebellum confirm their implication in the regulation of motor activity [32]. Although less characterized, CB2 expression, encoded by the *cnr2* gene, has also been reported in several tissues (including neural tissue), with predominant expression occurring on peripheral immune cells [30]. From a physiological point of view, the activation of cannabis receptors in the CNS modulates the adenylate cyclase activity, which in turn inhibits cyclic adenosine monophosphate accumulation, voltage-gated calcium and potassium channels and neurotransmitter release in presynaptic excitatory and inhibitory synapses [33]. For this reason, these receptors are indicated in many disorders affecting the brain, including several neurodegenerative disorders such as Huntington’s disease, multiple sclerosis and Alzheimer’s disease [34].

So far, the majority of scientific research on the effect of cannabinoids was based on *in vitro* approaches or on rodent models, but recently, the zebrafish (*Danio rerio*) has gained attractiveness as a powerful in vivo model for complementing and expanding the findings of these existing studies. The zebrafish is, nowadays, widely used as an animal model for pharmacological screening [35,36,37,38], owing to its excellent adaptation to laboratory conditions and its advantageous features already reviewed elsewhere [39,40,41,42,43,44]. In drug screenings, zebrafish are able to absorb small molecules across the skin from the surrounding water, at all stages of development [31]. For these reasons, zebrafish have already been used to investigate the effects of specific cannabinoid administration in numerous contexts, including addiction, anxiety, development, energy homeostasis and food intake, immune system function and learning and memory [31]. CB1 and CB2 have similar expression profiles in the zebrafish CNS compared to mammals, and CB1 shares a 70% protein sequence identity with its human homolog. Moreover, both CB1 and CB2 can be detected in zebrafish as early as the start of gastrulation stage [29]. Therefore, this cyprinid can be a favorable model organism to further illuminate evolutionary conserved biological mechanisms related to ECS [45].

A recent study shows that *cnr1* activation by exogenous endocannabinoids modulates locomotor activity in zebrafish larvae [46], probably acting on the hypothalamus, which is supposed to be the region involved in locomotion regulation in fishes [47]. Moreover, it has been demonstrated that knockdown of *cnr1* gene activity leads to defects in axonal growth and fasciculation [48]. Overall, cannabinoids were reported to affect the locomotor activity of zebrafish larvae by a biphasic response: locomotion stimulation at low doses and inhibition at high doses [24]. To our best knowledge, no studies have been published on the preclinical effects of cannabis’ full extract on the zebrafish model. Since cannabis consumers typically use the whole cannabis inflorescences, more attention should be paid to all the cannabinoids and their interaction, and not only to the main neuro-active constituents of cannabis, such as THC and CBD. Therefore, the aim of the present study was to investigate the effects of different doses of a cannabis full extract on zebrafish larvae. These effects were evaluated on egg hatching, larvae survival, locomotion behavior and mRNA expression of selected ECS-related genes.

## 2. Results

### 2.1. Embryo Development and Survival

**Figure 1:**
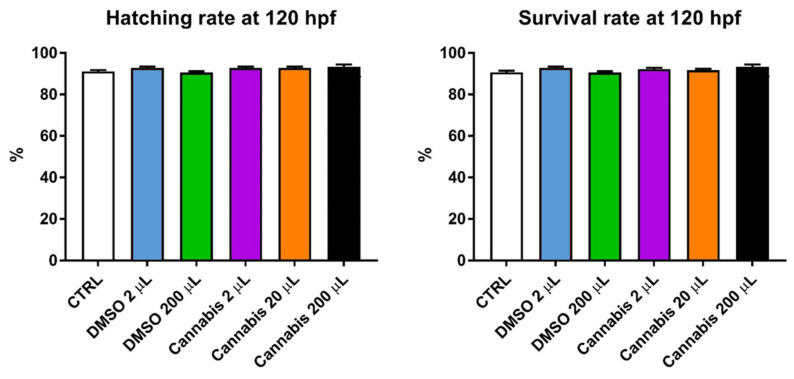
Fish hatching and survival rate at 120 h post-fertilization (hpf). Values are expressed as means (*n* = 90 per each group). The error bars show the standard error of the mean.

At 120 h post-fertilization (hpf), both embryo hatching and larvae survival rates were higher than 90% for all the treatments, and no significant differences were observed (Figure 1). Our results suggest that both dimethyl sulfoxide (DMSO) and cannabis treatments exhibited no toxicity to zebrafish at the concentrations used. In detail, the highest hatching rate was observed in the group treated with 200 μL of cannabis extract (93.89%), followed by the groups treated with 2 and 20 μL of cannabis extract and the group treated with 2 μL of DMSO (92.78%), the control group (91.11%) and, finally, the lowest hatching rate was detected in the DMSO 200 μL group (90.56%). Similarly, the highest survival rate was observed in the group treated with 200 μL of cannabis extract (93.33%), followed by the group treated with 2 μL of DMSO (92.78%), the groups treated with 2 and 20 μL of cannabis extract (92.22 and 91.67%, respectively), the control group (90.74%) and once again, the lowest survival rate was observed in the DMSO 200 μL group (90.56%).

### 2.2. Locomotion Behavior

**Figure 2:**
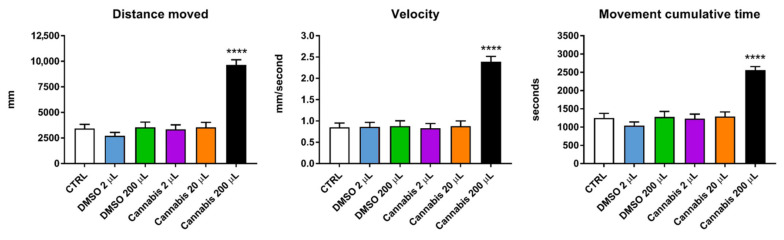
Locomotion behavior of untreated and treated larvae during the first 150 min. Values are expressed as means (*n* = 32 per each group) and the error bars show the standard error of the mean. Cannabis 200 µL group differs with all other groups per **** *p* ≤ 0.0001.

The locomotor behavior of the zebrafish larvae (distance moved, velocity and movement cumulative time) during the first 150 min of the test are reported in Figure 2. Compared to the control and DMSO treated groups, the larvae treated with cannabis at the highest dosage showed an increased locomotion, with greater velocity and higher movement cumulative time. Overall, these results showed that 200 µL per 100 mL of cannabis extract enhanced zebrafish larvae locomotor behavior.

**Figure 3:**
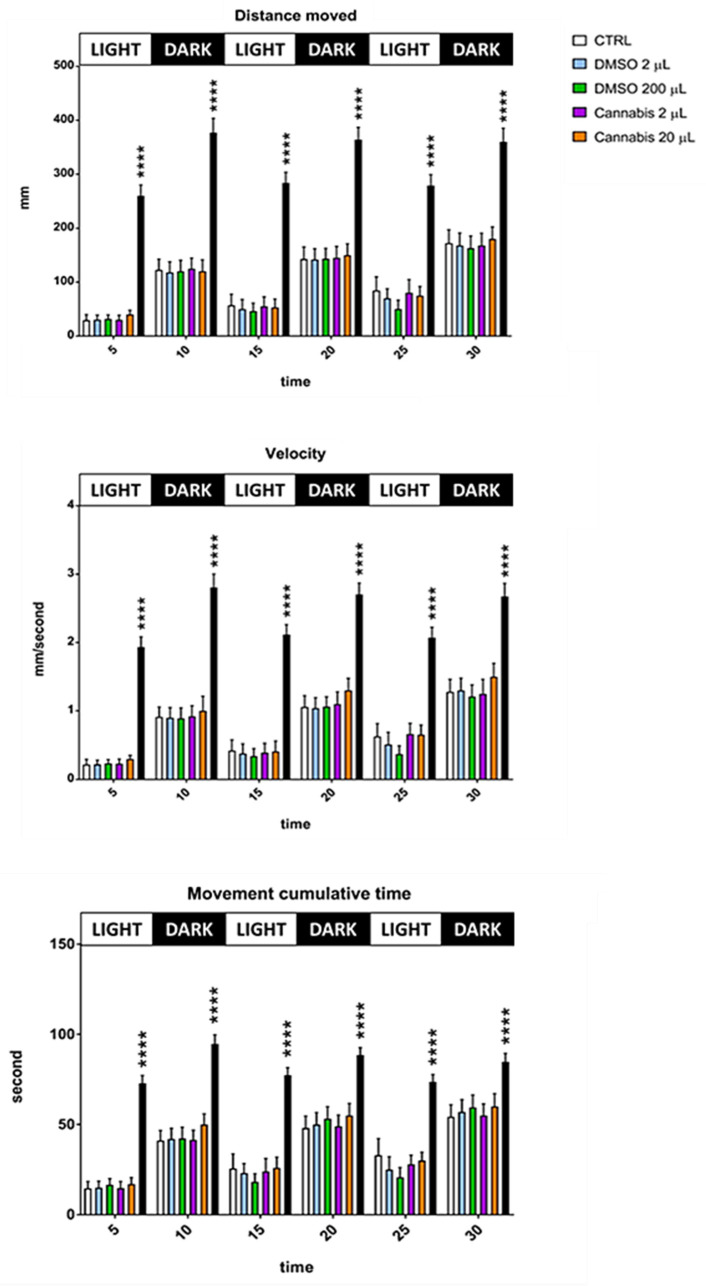
Locomotion performances of untreated and treated larvae during the 30 min of alternating light/dark cycles. Values are expressed as means (*n* = 32 per each group) and the error bars show the standard error of the mean. Cannabis 200 µL group differs with all other groups per **** *p* ≤ 0.0001.

Data on locomotion behavior during the alternating light/dark cycles are presented in Figure 3. As occurred in the first 150 min when the light was always on, during the light/dark cycles, the zebrafish larvae treated with the cannabis whole extract at the highest dosage traveled a greater distance at higher velocity, and spent more time in movement compared to the control and DMSO treated groups. These findings support the hypothesis that the cannabis full extract can cause an excitatory effect on zebrafish larval locomotion, even during changes in lighting intensity.

### 2.3. Analysis of Cannabinoid Receptors Expressions

**Figure 4:**
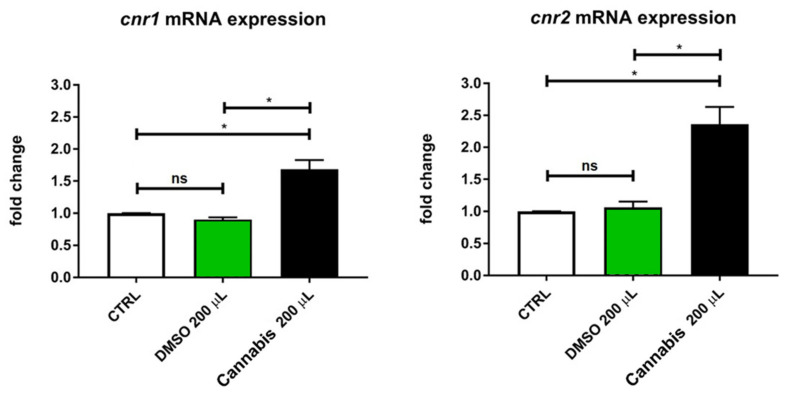
Quantitative real-time PCR analysis of *cnr1* and *cnr2* mRNA in untreated and in treated (DMSO or cannabis) larvae. Values are expressed as means (*n* = 90 per each group). The error bars show the standard error of the mean. ns *p* > 0.05, * *p* ≤ 0.05.

Expression analyses of mRNA of *cnr1* and *cnr2* cannabinoid receptors were performed on the cannabis treated group that showed locomotion behavior alterations (200 µL group) between its DMSO counterpart and with the control group. mRNA expression evaluation was obtained through real-time PCR experiments. Our results suggest that cannabis treatment significantly affected both the zebrafish cannabinoid receptors *cnr1* and *cnr2* by increasing their expressions (see Figure 4). In particular, *cnr1* and *cnr2* expression of cannabis-treated larvae was 70 and 130% higher than the controls, respectively.

## 3. Discussion

Plants provide a wide array of opportunities for discovering new drugs and natural compounds. Their use in human medicine requires an evaluation of toxicity to figure out possible exposure risks [49]. The gold standard for predictive analysis of chemical risks to humans remains vertebrate toxicity studies [35]. In this context, zebrafish larvae have become a tool widely used to assess the toxic effects of chemicals, drugs, and natural compounds [36,38]. The present study evaluated, for the first time, the effects of a whole cannabis extract diluted in DMSO on zebrafish development, locomotion and mRNA expression of some ECS-related genes (*cnr1* and *cnr2*). In accordance with Hallare et al. [50], the results confirmed that a concentration of DMSO up to 0.2% (*v*/*v*) has no negative effects on zebrafish hatching and survival rate. To this regard, Hallare et al. [50] reported that DMSO concentrations up to 1% (*v*/*v*) did not cause adverse effects on zebrafish development and survival. Indeed, both hatching rates and survival rates were similar among controls, DMSO and cannabis extract treated groups. Hatching rate represents a widely used endpoint on toxicological studies involving zebrafish larvae and, generally, embryos started to hatch at 48 hpf and finished at 96 hpf [51]. The survival rate of control group (91%) was very similar to the value reported by Hernandez et al. [52] for untreated zebrafish larvae of the same age (120 hpf).

Zebrafish locomotor performances were greatly affected by the highest dose of cannabis extract, both under standard light conditions and under light/dark cycles. Zebrafish larvae typically exhibit a higher locomotion behavior during dark periods and slower movements under bright conditions [53]. In this study, zebrafish larvae treated with 200 µL of cannabis whole extract traveled a greater distance at higher velocity and spent more time in movement compared to controls and DMSO treated groups. Several authors have already described this excitatory effect on zebrafish larvae locomotion by using low doses of active substances extracted from cannabis. In particular, THC exposure for 96 h (the same exposure period as the current study) caused significant hyperactivity of the zebrafish larvae when administered at doses ranging from 0.3 mg/L [28] to 1.2 mg/L [24]. Similarly, CBD significantly stimulated locomotor activity at concentrations ranging from 0.02 to 0.3 mg/L [28,54]. In contrast, doses of THC higher than 1.25 mg/L [25,28] or doses of CBD above 10 mg/L [8,55] significantly reduced the locomotor activity of the zebrafish larvae. These results are consistent to those observed in mice, where a stimulation of locomotor activity by THC at low concentrations was observed, and a suppression at higher concentrations [56]. In the present study, the concentration of THC and CBD was extremely low compared to the above-mentioned studies, but the observed effects on locomotion are probably related not only to the presence of the main neuro-active cannabis compounds (notably THC and CBD) but to the synergic effect of all the cannabis inflorescence constituents. Moreover, the cannabis extract solution led to an increase in both mRNA’s cannabinoids receptors (*cnr1* and *cnr2*), in a manner consistent with behavioral outcomes. Similarly, the exposure of zebrafish larvae to an endocannabinoid (anandamide) significantly upregulated the *cnr1* gene expression at all the developmental stages analyzed: 72 hpf, 7 and 15 days post-fertilization (dpf) [57]. The treatment of both exogenous and endogenous cannabinoids has also been implicated in elevating *cnr1* mRNA levels in a variety of cell types and tissues [58]. As already described, *cnr1* mRNA co-localizes in the hypothalamus with an enzyme involved in dopamine synthesis [59]. Therefore, this evidence suggests that cannabinoids’ receptors, together with the ECS, modulate the dopamine transmission [60] and the hypothalamus function, regulating locomotion [47].

## 4. Materials and Methods

The present study was carried out at the zebrafish facility of the Veterinary Sciences Department of the University of Pisa (Pisa, Italy), in collaboration with IRCCS Fondazione Stella Maris (Scientific Hospitalization and Care Institute, Calambrone, Italy) and the PlantLab of the Institute of Life Sciences of the Scuola Superiore Sant’Anna (Pisa, Italy). The study was also conducted in accordance with the directive 2010/63/EU on the protection of animals used for scientific purposes.

### 4.1. Plant Material and Preparation of the Cannabis Extract

Seeds of *Cannabis sativa* var. “Zenit” (Naturfibre S.r.l., Casciana Terme Lari, Pisa, Italy) were sown in 160 loam wells and incubated at 24 °C for two days in dark conditions. After germination, homogeneous seedlings were selected and transplanted into individual pots (⌀33 cm) filled with a biological substrate (Brill Ortopack Bio; Agrochimica S.p.A., Bolzano, Italy) composed by blond peat (fraction 0–5 mm), coconut fiber (light fraction), and black peat (fraction 0–6 mm). The substrate was characterized as follows: apparent density of 270–320 g/L, air volume of 20–25%, water retention capacity of 5.8 g/g, pH 5.5–6.5; electrical conductivity <1 mS/cm; N 365 mg/L; P 125 mg/L; K 167.5 mg/L; Mg 12 mg/L; Fe 15 mg/L; S 38 mg/L. Plants were grown in a glasshouse located in central Italy (San Giuliano Terme, Pisa) from June to September, under natural temperature and light conditions (mean air temperature averaged 30 °C and 20 °C during night and day time, respectively, and the light intensity ranged between 100,000 and 150,000 lux). Plants were irrigated daily by aspersion and fertilized every 20 days during the vegetative stage by adding a chemical fertilizer (NPK ratio of 8–12–10) to the watering solution. Plant fertilization and irrigation were interrupted at the beginning of flowering (second week of August) and one week before harvest (on September 15). Once collected, flowers and leaves were mixed and dried in a naturally ventilated room at 20 °C for two weeks, avoiding exposure of the material to light sources.

The dried plant material was then divided into three different subsamples which were processed to extract the phytocomplex in a resin form, and then characterized for the THC and CBD content by gas chromatography–mass spectrometry. Details of the extraction and quantification protocols adopted are fully described by Caprioglio et al. [61]. All the biological tests performed in the current study were conducted by using only the cannabis resin subsample characterized by the lowest THC level. This selected cannabis extract, which weighed 907 mg, was solubilized by adding 80 mL of DMSO. The organic solvent was added to allow the solubilization and the dispersion of lipophilic compounds, as cannabis extract, into the test media prior to its exposure to the zebrafish larvae. Finally, the DMSO–cannabis extract was diluted in egg water to be used for the in vivo tests. The THC and CBD concentration of the final solution was determined prior to egg treatments.

### 4.2. Fish Management

Adult zebrafish (wild-type, AB strain) were maintained and mated according to standard procedures [62]. The zebrafish eggs necessary for the test were obtained from natural spawning and, once collected, viable eggs were incubated at 28 °C for 24 h into petri dishes containing egg water solution (60 mg of “Instant Ocean” sea salts per 1 L of distilled water).

### 4.3. Experimental Design

At 24 hpf, 540 embryos were selected and randomly divided in 18 groups of 30 individuals each. Since several authors [50,63] reported a possible dose-dependent effect of DMSO on fish embryo development and hatching rate, the effect of the exposure to DMSO solution was also studied. Overall, six groups with three replicates each were prepared: the control group (no exposition to DMSO or cannabis extract), the DMSO treated groups (addition of 2 or 200 μL of DMSO per 100 mL egg water) and the cannabis treated groups (addition of 2, 20 or 200 μL of DMSO-cannabis extract per 100 mL egg water). The cannabis extract solution used for the in vivo test contained 1.73 nM of THC and 22.3 nM of CBD at the highest dose (200 μL). Once the different groups, with their replicates, were prepared, embryos were repositioned in the incubator until 5 dpf. According to Akhtar et al. [24], the exposures to DMSO or cannabis in the treated groups can be defined as chronic (96 h of exposition starting at 24 hpf).

### 4.4. Measurements and Analysis

During the experimental period, egg hatching rate and embryo survival were monitored daily. Distance moved, velocity and movement cumulative time were measured as zebrafish locomotor behavior indicators on untreated and treated larvae at 5 dpf. To this purpose, the DanioVision system and EthoVision XT12 software (Noldus Information Technology, Wageningen, The Netherlands) were used as previously described by Licitra et al. [36]. Together with 300 µL of the solution treatment for a single larva, 32 individuals of each group were transferred from the rearing dishes to a 96 multiwell plate and placed into the DanioVision system for 1 h of acclimatization. Finally, larvae locomotor behavior was monitored for 180 min total—150 min with the light on and 30 min with alternating 5 min light on and 5 min light off (darkness), for a total of three light on/light off cycles.

### 4.5. qRT-PCR

Total RNA was extracted from larvae at 5 dpf using the Quick RNA miniprep kit (ZymoResearch, Irvine, USA), according to the manufacturer’s instruction. cDNA and qRT-PCR were performed as described by Brogi et al. [64]. Relative mRNA expression was quantified using the comparative ΔCt method and expressed as 2^−ΔΔCt^. The results obtained were normalized to the expression of the housekeeping gene, β-actin (ENSDARG00000037746). The mean of the controls was set equal to one. The sequences of the primers used are listed in Supplementary Materials Table S1. Each assay was performed in triplicate, and 90 larvae per group were analysed.

### 4.6. Statistical Analysis

All data were analyzed by Analysis of Variance (ANOVA), and the observed means were compared by Tukey–Kramer HSD test. All statistical analyses were performed using GraphPad Prism (GraphPad Software, Inc., San Diego, CA, USA) and differences between treatments were considered significant for *p* ≤ 0.05.

## 5. Conclusions

In conclusion, the chronic exposure of zebrafish larvae to a whole extract of cannabis affected zebrafish behavior as already observed for rodents [56]. In fact, the use of a cannabis-derived product characterized by low content of THC and CBD caused an enhancement in the locomotor performances of zebrafish larvae. The treatment did not cause adverse effects on embryo development in terms of hatching rates and larvae survival, which are the major outcome measures for toxicological screening in zebrafish pharmacological studies. Therefore, these findings open up the use of whole extract of cannabis for applications in the biomedical field as well as in several animal sectors, notably aquaculture.

## Data Availability

Data are contained within the article and Supplementary Material.

## Supplementary Materials

The following are available online at https://www.mdpi.com/article/10.3390/ph14121224/s1, Table S1: Primer sequences for qRT-PCR. Graphical abstract was created with BioRender.com.
