# Supplementary material for: In Vivo Evaluation of Cannabis sativa Full Extract on Zebrafish Larvae Development, Locomotion Behavior and Gene Expression
Source: Pharmaceuticals (Basel). 2021 Nov 25;14(12):1224. doi: 10.3390/ph14121224 (PMC8705266; doi:10.3390/ph14121224)
Supplement: Supplementary file 1 [file pharmaceuticals-14-01224-s001.zip › pharmaceuticals-1472290-supplementary.pdf]

Table S1: qPCR primers used for gene expression analysis

Gene	Nucleotide Sequence
β-actin-Fw	5′-GCAGAAGGAGATCACATCCCTGGC-3′
β-actin-Rv	5′-CATTGCCGTCACCTTCACCGTTC-3′
cnr1-Fw	5′- GACACCTGCGCGAACTATGT-3′
cnr1-Rv	5′- TCGTCGTAGCCGATGTCATT-3′
cnr2-Fw	5’- GTGGGAAGCCTTTTGCTGAC-3′
cnr2-Rv	5’- CTCCACCCCATCAAAGGGAG-3′
